# Hypoglycaemia risk with insulin degludec compared with insulin glargine in type 2 and type 1 diabetes: a pre-planned meta-analysis of phase 3 trials

**DOI:** 10.1111/dom.12032

**Published:** 2012-12-03

**Authors:** R E Ratner, S C L Gough, C Mathieu, S Del Prato, B Bode, H Mersebach, L Endahl, B Zinman

**Affiliations:** 1 Division of Endocrinology, Department of Medicine, Georgetown University School of MedicineWashington, DC, USA; 2 Oxford Centre for Diabetes, Endocrinology and Metabolism and NIHR Oxford Biomedical Research CentreOxford, UK; 3 University Hospitals (UZ) Leuven, KU LeuvenLeuven, Belgium; 4 University of PisaPisa, Italy; 5 Atlanta Diabetes AssociatesAtlanta, GA, USA; 6 Novo NordiskSøborg, Denmark; 7 University of Toronto, Mount Sinai HospitalToronto, ON, Canada

**Keywords:** confirmed hypoglycaemia, insulin degludec, insulin glargine, nocturnal hypoglycaemia, severe hypoglycaemia

## Abstract

**Aim:**

Hypoglycaemia and the fear of hypoglycaemia are barriers to achieving normoglycaemia with insulin. Insulin degludec (IDeg) has an ultra-long and stable glucose-lowering effect, with low day-to-day variability. This pre-planned meta-analysis aimed to demonstrate the superiority of IDeg over insulin glargine (IGlar) in terms of fewer hypoglycaemic episodes at equivalent HbA1c in type 2 and type 1 diabetes mellitus (T2DM/T1DM).

**Methods:**

Pooled patient-level data for self-reported hypoglycaemia from all seven (five in T2DM and two in T1DM) randomized, controlled, phase 3a, treat-to-target trials in the IDeg clinical development programme comparing IDeg once-daily (OD) vs. IGlar OD were analysed.

**Results:**

Four thousand three hundred and thirty subjects (2899 IDeg OD vs. 1431 IGlar OD) were analysed. Among insulin-naïve T2DM subjects, significantly lower rates of overall confirmed, nocturnal confirmed and severe hypoglycaemic episodes were reported with IDeg vs. IGlar: estimated rate ratio (RR):0.83[0.70;0.98]_95%__CI_, RR:0.64[0.48;0.86]_95%__CI_ and RR:0.14[0.03;0.70]_95%__CI_. In the overall T2DM population, significantly lower rates of overall confirmed and nocturnal confirmed episodes were reported with IDeg vs. IGlar [RR:0.83[0.74;0.94]_95%__CI_ and RR:0.68[0.57;0.82]_95%__CI_). In the T1DM population, the rate of nocturnal confirmed episodes was significantly lower with IDeg vs. IGlar during maintenance treatment (RR:0.75[0.60;0.94]_95%__CI_). Reduction in hypoglycaemia with IDeg vs. IGlar was more pronounced during maintenance treatment in all populations.

**Conclusions:**

The limitations of this study include the open-label design and exclusion of subjects with recurrent severe hypoglycaemia. This meta-analysis confirms that similar improvements in HbA1c can be achieved with fewer hypoglycaemic episodes, particularly nocturnal episodes, with IDeg vs. IGlar across a broad spectrum of patients with diabetes.

## Introduction

Improving glycaemic control through timely and intensive diabetes treatment reduces the risk of developing complications in patients with diabetes 1,2. With appropriate titration, insulin therapy is highly efficacious but is associated with hypoglycaemia. The undesirable effects of hypoglycaemia on patient's well-being, daily routine and lifestyle can pose a significant burden to patients and the community through the loss of productivity and the increase in treatment costs 3,4. Hypoglycaemia and the fear of hypoglycaemia are significant limiting factors in achieving glycaemic control with insulin because the negative physiological, social and psychological consequences of these episodes challenge the willingness of physicians and patients to increase the dose 5. Innovations in insulin therapy have led to a lower risk of hypoglycaemia with current insulin treatments 6,7; however, the residual risk of hypoglycaemia continues to limit the ability to safely achieve normoglycaemia 8.

Insulin degludec (IDeg) is an ultra-long-acting basal insulin in clinical development for the treatment of type 2 and type 1 diabetes mellitus (T2DM and T1DM). Soluble multi-hexamers are formed in the subcutaneous tissue upon injection, creating a depot from which monomers are slowly and continuously absorbed into the circulation 9. This results in a flatter and more stable pharmacokinetic and pharmacodynamic profile, with less variability in glucose-lowering activity compared with insulin glargine (IGlar) 10,11. In two exploratory phase 2 trials (one involving T2DM subjects and the other involving T1DM subjects), subjects reported fewer hypoglycaemic episodes with IDeg compared with IGlar while achieving similar glycaemic control 12,13.

The aim of this pre-planned meta-analysis was to show the superiority of IDeg over IGlar in terms of hypoglycaemic episodes at equivalent HbA1c using pooled, individual patient-level data from all phase 3 trials in the IDeg clinical development programme, as discussed in advance with the regulatory authorities. The treat-to-target trial design was adopted for all of the trials included in this meta-analysis and comparable titration algorithms were applied in an attempt to achieve similar glycaemic goals.

## Methods

### Trial Design, Participants and Hypoglycaemia Assessments

This pre-planned meta-analysis included pooled patient-level data from all seven trials in the IDeg development programme that compared IDeg once daily (OD) vs. IGlar OD (five T2DM trials and two T1DM trials) (Appendix Table A1) 14–21. In Trials 3668 (T2DM) and 3770 (T1DM), two different OD dosing regimens of IDeg were evaluated. The flexible dosing arms in these two trials, with prespecified alternating morning and evening dosing, were excluded from this meta-analysis because they did not reflect the intended clinical use of IDeg.

All trials were randomized, controlled, open-label, multicentre, phase 3a, treat-to-target trials of 26 or 52 week's duration (Appendix Table A1). Key recruitment criteria included the following elements: no history of recurrent severe hypoglycaemia (i.e. having no more than one severe episode in the preceding 12 months) and baseline HbA1c 7.0–10.0% (in T2DM) or ≤10.0% (in T1DM). Similar concomitant oral antidiabetic treatments were allowed. Insulin doses were adjusted to achieve self-measured prebreakfast blood glucose targets calibrated to plasma glucose values of 4.0 to <5.0 mmol/l (>70 to <90 mg/dl), using the same titration guideline for both basal insulins 14–21. This resulted in similar glycaemic control between IDeg and IGlar in all trials, confirming non-inferiority of IDeg to IGlar (applying an HbA1c margin of 0.4%) 22. Self-reported hypoglycaemic episodes were contemporaneously recorded in daily patient diaries. Definitions of hypoglycaemic episodes are listed in Table 1.

**Table 1:** Classification of hypoglycaemic episodes

Hypoglycaemic Episode Category	Definition
Treatment-emergent hypoglycaemic episodes	Episodes occurring between first trial drug exposure and 7 days after last trial drug exposure
Severe hypoglycaemic episodes	Episodes during which the subject required assistance in administering carbohydrates, glucagon or other resuscitative actions
Confirmed hypoglycaemic episodes	Includes episodes confirmed by a plasma glucose of <3.1 mmol/l (56 mg/dl) and severe episodes
	This threshold gives a fair balance between glucose levels at which counter-regulatory mechanisms are triggered and levels at which patients typically report symptoms. This also adheres to the European Medicines Agency (EMA) guidelines 31
Nocturnal confirmed hypoglycaemic episodes	Confirmed episodes occurring between 00:01 h and 05:59 h (both inclusive)

### Statistical Analysis

The meta-analysis plan was based on prior discussions with and subsequent review by regulatory authorities prior to the unblinding of data for the individual trials. The reviewer's comments were addressed with additional analyses, including analyses for hypoglycaemia during the maintenance phase (after stable glycaemic control and stable insulin dose had been achieved following active titration). Data for insulin-naïve T2DM subjects, all T2DM subjects and T1DM subjects were combined in the primary analysis and further analysed as separate populations. All randomized subjects were analysed following the intent-to-treat principle.

The primary endpoint for this meta-analysis was overall confirmed hypoglycaemia (defined in Table 1). Nocturnal confirmed and severe hypoglycaemic episodes (subsets of overall confirmed episodes) were evaluated separately. Changes in hypoglycaemic rates across the study timeline were compared between treatments during the titration period (0–15 weeks of treatment) and the maintenance period (from 16 weeks to the end of treatment). Treatment-emergent hypoglycaemic episodes were counted for each subject, divided by exposure time (as an offset in the model), and analysed using a negative binomial regression model adjusted for differences across trials, diabetes type, antidiabetic therapy at screening, sex, geographical region and age. The negative binomial model (an extension of the Poisson model) allows for heterogeneity between subjects arising from within-subject correlation. Sensitivity analyses were performed (one without covariates and another using trial by treatment interaction as a random effect). Hypoglycaemic rates were expressed as the number of episodes per patient-year of exposure (PYE). Treatment differences were reported as estimated rate ratios (RRs) of IDeg/IGlar, with [95% CI].

### Role of the Funding Source

Novo Nordisk contributed to the study design, statistical analyses, data interpretation, manuscript preparation and the decision to submit this manuscript for publication. All of the authors had access to trial data and took full responsibility for the content of the manuscript and the decision to submit it for publication.

## Results

### Trial Characteristics and Study Participants

This meta-analysis comprised 4330 subjects: 2899 randomized to IDeg OD and 1431 randomized to IGlar OD. Withdrawal rates (16.6% [IDeg OD] and 13.8% [IGlar OD]), baseline characteristics and demographics were similar between groups (Appendix Table A2). Among previous insulin users, 30–40% of T2DM subjects and 60–70% of T1DM subjects had previously been treated with IGlar. Hypoglycaemia occurred at a notably lower rate in T2DM subjects than in T1DM subjects (Table 2).

**Table 2:** Rate (number of episodes per patient year of exposure) and incidence (% of subjects) of hypoglycaemic episodes in subjects with type 2 or type 1 diabetes mellitus*

Trial	3579		3672		3586		3668		3582		3583		3770	
Population	T2DM		T2DM		T2DM		T2DM		T2DM		T1DM		T1DM	
Treatment*	IDeg	IGlar	IDeg	IGlar	IDeg	IGlar	IDeg	IGlar	IDeg	IGlar	IDeg	IGlar	Igeg	IGlar
No. of subjects	773	257	228	229	289	146	228	230	744	248	472	157	165	164
Number of overall confirmed hypoglycaemic episodes per PYE(% subjects)														
Entire treatment period	1.5	1.8	1.2	1.4	3.0	3.7	3.6	3.5	11.1	13.6	42.5	40.2	88.3	79.7
	(46.5)	(46.3)	(28.5)	(30.7)	(50.0)	(53.4)	(43.8)	(49·3)	(80.9)	(82.1)	(95.6)	(95.5)	(99.4)	(96.9)
Titration period	1.4	1.4	1.1	1.0	3.1	3.4	3.3	3.3	13.0	14.9	53.1	48.6	92.9	82.7
	(23.6)	(23.0)	(18.4)	(19.8)	(41.1)	(43.7)	(36.4)	(41.4)	(71.2)	(71.2)	(93.3)	(94.6)	(98.7)	(96.1)
Maintenance period	1.6	2.1	1.4	2.1	2.4	3.9	3.6	3.7	10.1	13.0	37.3	36.2	76.5	75.2
	(41.2)	(42.9)	(18.4)	(21.4)	(28.7)	(33.1)	(26.3)	(32.2)	(72.3)	(72.8)	(93.8)	(93.2)	(90.5)	(89.0)
Number of nocturnal confirmed hypoglycaemic episodes per PYE (% subjects)														
Entire treatment period	0.3	0.4	0.2	0.3	0.8	1.2	0.6	0.7	1.4	1.8	4.4	5.9	9.6	10.0
	(13.8)	(15.2)	(6.1)	(8.8)	(20.4)	(24.0)	(10.6)	(21.4)	(39.6)	(47.4)	(72.2)	(74.0)	(73.3)	(72.7)
Titration period	0.2	0.2	0.2	0.2	0.8	1.2	0.4	0.7	1.7	1.8	5.3	7.2	10.4	10.1
	(4.9)	(4.4)	(4.9)	(4.2)	(16.0)	(19.0)	(6.8)	(16.2)	(25.9)	(28.4)	(54.6)	(60.1)	(69.1)	(66.5)
Maintenance period	0.3	0.5	0.2	0.5	0.7	1.3	0.9	0.9	1.3	1.9	3.9	5.2	7.7	9.5
	(12.3)	(14.2)	(2.9)	(7.1)	(10.7)	(12.2)	(7.8)	(11.5)	(29.7)	(37.0)	(60.3)	(64.2)	(48.6)	(49.7)
Number of severe hypoglycaemic episodes per PYE (% subjects)														
Entire treatment period	0.003	0.02	0	0	0	0.01	0.02	0.02	0.06	0.05	0.2	0.2	0.4	0.5
	(0.3)	(1.9)				(0.7)	(0.9)	(0.9)	(4.5)	(4.4)	(12.3)	(10.4)	(12.7)	(9.9)

IDeg, insulin degludec; IGlar, insulin glargine; PYE, patient-year of exposure; T2DM, type 2 diabetes mellitus; T1DM, type 1 diabetes mellitus.

* IDeg and IGlar were both injected once daily.

### Hypoglycaemia in Insulin-naïve T2DM Subjects

Insulin-naïve subjects experienced significantly lower rates of overall confirmed episodes (RR 0.83[0.70;0.98]) and nocturnal confirmed episodes (RR 0.64[0.48;0.86]) with IDeg than with IGlar across the entire treatment period (Tables 2 and 3). The majority of these overall confirmed and nocturnal confirmed episodes were symptomatic (53.3%, 69.5% [IDeg]; 58.4% and 73.6% [IGlar]). The rates of overall confirmed episodes (RR 0.72[0.58;0.88]) and nocturnal confirmed episodes (RR 0.51[0.36;0.72]) during the maintenance period were even lower with IDeg than with IGlar, compared with the titration period. A significantly lower rate of severe episodes (RR 0.14[0.03;0.70]) was reported with IDeg than with IGlar across the entire treatment period (Tables 2 and 3).

**Table 3:** Hypoglycaemia risk in subjects with type 2 or type 1 diabetes mellitus

	Estimated Rate Ratio* [95% Confidence Interval]		
Population	Insulin-naïve T2DM	T2DM	T1DM
Number of subjects			
IDeg†	1290	2262	637
IGlar†	632	1110	321
Overall confirmed hypoglycaemic episodes			
Entire treatment period	0.83 [0.70;0.98]‡	0.83 [0.74;0.94]‡	1.10 [0.96;1.26]
Titration period	0.95 [0.76;1.18]	0.92 [0.80;1.05]	1.14 [1.00;1.30]
Maintenance period	0.72 [0.58;0.88]‡	0.75 [0.66;0.87]‡	1.02 [0.88;1.19]
Nocturnal confirmed hypoglycaemic episodes			
Entire treatment period	0.64 [0.48;0.86]‡	0.68 [0.57;0.82]‡	0.83 [0.69;1.00]
Titration period	0.90 [0.60;1.36]	0.81 [0.64;1.02]	0.88 [0.72;1.08]
Maintenance period	0.51 [0.36;0.72]‡	0.62 [0.49;0.78]‡	0.75 [0.60;0.94]‡
Severe hypoglycaemic episodes			
Entire treatment period	0.14 [0.03;0.70] ‡	0.81 [0.42;1.56]	1.12 [0.68;1.86]

IDeg, insulin degludec; IGlar, insulin glargine; T2DM, type 2 diabetes mellitus; T1DM, type 1 diabetes mellitus.

* Estimated rate ratio: IDeg/IGlar.

† IDeg and IGlar were both injected once daily.

‡ Significantly lower risk with insulin degludec based on 95% confidence interval.

### Hypoglycaemia in All T2DM Subjects

Significantly lower rates of overall confirmed and nocturnal confirmed episodes were reported with IDeg than with IGlar in the overall T2DM population across the entire treatment period, and these differences were more apparent during the maintenance phase (Tables 2 and 3). The majority of these overall confirmed and nocturnal confirmed episodes were symptomatic (71.6 and 80.2% [IDeg]; 73.6 and 83.0% [IGlar]). The rate of severe episodes was lower with IDeg than with IGlar across the entire treatment period, although this difference was not statistically significant (Tables 2 and 3).

### Hypoglycaemia in T1DM Subjects

The majority of the overall confirmed and nocturnal confirmed episodes in T1DM subjects were symptomatic (76.1 and 84.9% [IDeg]; 73.1 and 79.0% [IGlar]). There was no significant difference between treatments in the rate of overall confirmed episodes across the entire treatment period, although it was slightly higher with IDeg than with IGlar (Tables 2 and 3). The rate of nocturnal confirmed episodes across the entire treatment period was 17% lower with IDeg than with IGlar (not statistically significant), whereas the rate of nocturnal confirmed episodes during the maintenance period (RR 0.75[0.60;0.94]) was significantly lower with IDeg than with IGlar. The rate of severe episodes across the entire treatment period was slightly, but not significantly, higher with IDeg than with IGlar (Tables 2 and 3)

### Hypoglycaemia in the Pooled T2DM and T1DM Population

Subjects in the pooled population experienced significantly lower rates of overall confirmed episodes (RR 0.91[0.83;0.99]) and nocturnal confirmed episodes (RR 0.74[0.65;0.85]) with IDeg than with IGlar across the entire treatment period (the full analysis of the pooled T2DM and T1DM population is presented in Appendix Table A3).

### Robustness of Results

**Figure 1:**
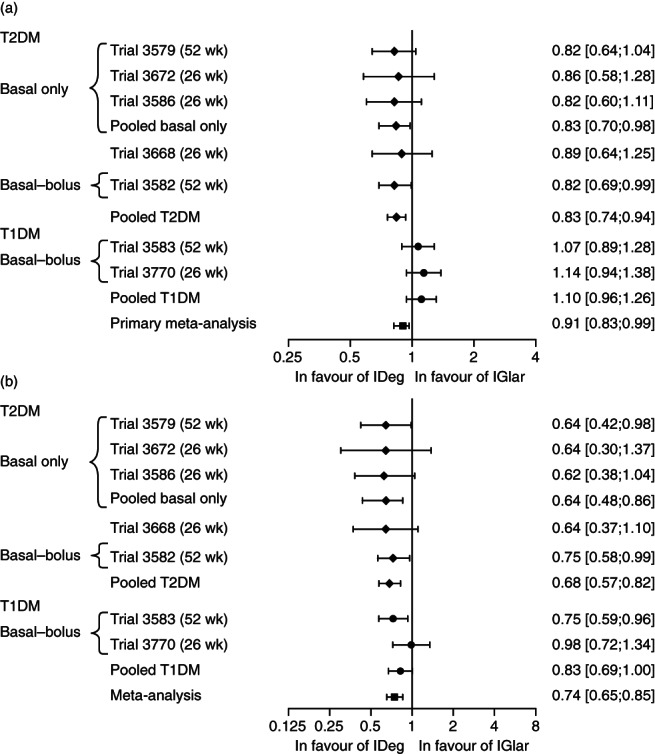
Estimated rate ratios (IDeg/IGlar) and 95% confidence intervals of (a) overall confirmed hypoglycaemic episodes and (b) nocturnal confirmed hypoglycaemic episodes for individual trials.

A high degree of consistency in the relative rate of hypoglycaemia (IDeg vs. IGlar) was demonstrated in the Forest Plots, both among the five trials involving T2DM subjects and, separately, between the two trials involving T1DM subjects (Figure 1). A high degree of consistency in the rate of nocturnal hypoglycaemia was observed across all trials. Similar trends were observed in the titration and maintenance periods, with less discrimination between treatments during the titration period. The random effects approach (used in meta-analyses with a high degree of heterogeneity) produced similar results to the fixed-effect approach for confirmed hypoglycaemia in the pooled population (RR 0.91 [0.82;1.00] vs. RR 0.91 [0.83;0.99]; Appendix Table A3), thus emphasizing the consistency and robustness of the meta-analysis. The results were also robust with respect to the choice of covariates, as the sensitivity analysis fitting the model without covariates (other than treatment, type of diabetes and trial) produced a similar estimate to that of the model adjusting for age, sex and anti-diabetic therapy at screening, in addition to trial treatment, type of diabetes and trial (RR 0.93 [0.85;1.02] vs. RR 0.91 [0.83;0.99]). These sensitivity evaluations were performed in relation to the analysis of the pooled T2DM and T1DM population as that was the primary analysis of this study.

## Discussion

Two major strengths of this meta-analysis are the inclusion of all phase 3 trials comparing IDeg OD to IGlar OD, and its pre-planned design. The results of this meta-analysis further contribute to the evaluation of the risk-benefit profile of IDeg 22, as they enhance the findings of the individual trials: treatment with IDeg is associated with a lower risk of hypoglycaemia, particularly nocturnal hypoglycaemia, compared with IGlar, at a similar level of glycaemic control. The sensitivity analyses showed that the estimated RR is fairly independent of baseline characteristics and suggests that this benefit should apply to a broad population.

The lower rate of hypoglycaemia, particularly nocturnal hypoglycaemia, observed with IDeg across trials is likely a consequence of its ultra-long and stable pharmacokinetic profile, and lower day-to-day variability in glucose-lowering action 23,24. The lower day-to-day variation in pharmacodynamic action observed with IDeg compared with IGlar may provide a more consistent and predictable insulin response that likely contributed to the consistent findings of this meta-analysis 23.

Hypoglycaemia stems from an excess of insulin action relative to endogenous and exogenous glucose supply. Current basal–bolus therapy consists of rapid-acting bolus insulin that provides mealtime coverage, and basal therapy that provides coverage in the post-absorptive and fasting states. Nocturnal hypoglycaemic episodes are typically unrelated to the use of bolus insulin; hence, the rate of nocturnal episodes provides the most relevant standard of comparison for basal insulin preparations. Therefore, it is of interest to observe that the largest and most consistent differences in the rate of hypoglycaemia between IDeg and IGlar were observed during the nocturnal period, when glycaemic control is primarily affected by basal insulin and less so by bolus insulin (used at mealtimes in Trials 3582, 3583 and 3770). This observation is further supported by the higher rates of overall confirmed hypoglycaemia observed with both IDeg and IGlar in the trials that used the basal–bolus treatment (including T1DM and T2DM trials) compared with trials that used the basal-only therapy (T2DM trials).

Although insulin-naïve T2DM subjects reported the fewest hypoglycaemic episodes, the lower rate of hypoglycaemia for IDeg compared with IGlar is noteworthy because the fear of hypoglycaemia is a major impediment to initiating the insulin treatment that will ultimately be required by many T2DM patients as the disease progresses 25. The results of the individual trials showed that subjects who initiated basal insulin therapy with IGlar can, after initial titration, expect 2.1 confirmed hypoglycaemic episodes per year, of which 0.5 episodes per year occur at night (Table 2; Trial 3579; maintenance period). This means that for every 100 subjects who initiate treatment with IDeg instead of IGlar, and are treated for 1 year, a total of 50 overall confirmed hypoglycaemic episodes, of which 20 are nocturnal confirmed episodes, will be avoided (calculated using the estimated 25% reduction in confirmed hypoglycaemic episodes and 38% reduction in nocturnal confirmed episodes in the maintenance period for the pooled T2DM population, respectively). Subjects treated with IGlar experienced 0.02 severe hypoglycaemic episodes per year across the entire treatment period (Table 2; Trial 3579); hence, for every 100 subjects treated with IDeg instead of IGlar for 1 year, two severe hypoglycaemic episodes will be avoided (calculated based on the estimated 86% reduction in severe hypoglycaemia).

The corresponding numbers for confirmed hypoglycaemia in T2DM subjects on a basal–bolus regimen are much higher: for every 100 subjects treated with IDeg instead of IGlar for 1 year, a total of 326 overall confirmed hypoglycaemic episodes, of which 71 are nocturnal confirmed episodes, will be avoided [calculated using the observed rates with IGlar in the maintenance period (Table 2; Trial 3582) and the estimated 25% reduction in overall confirmed episodes, and 38% reduction in nocturnal confirmed episodes in the maintenance period for the pooled T2DM population].

For the T1DM population, significantly fewer nocturnal hypoglycaemic episodes were reported with IDeg than with IGlar during the maintenance period. Subjects treated with IGlar can expect 5.2 nocturnal confirmed hypoglycaemic episodes per year (Table 2; Trial 3583; maintenance period); hence, for every 100 subjects treated with IDeg instead of IGlar for 1 year, 130 nocturnal confirmed episodes will be avoided in this population once the initial titration phase has been completed (calculated based on the estimated 25% reduction in nocturnal confirmed hypoglycaemic episodes during the maintenance period for the T1DM population).

The blinding of investigators and subjects to treatment identity had been considered during the designing of the trials, but this was found to be extremely difficult to carry out because of the differences between the insulin delivery devices used. Hence, the open-label design of the trials is a limitation that could have resulted in a reporting bias. If such a reporting bias had been present, it would most likely have been constant over time. It is likely that the more pronounced benefit of IDeg during the maintenance period reflects the learning curve for optimal titration associated with the use of a new insulin preparation among trial participants rather than a differential reporting bias that changes over time.

The same titration algorithm was used consistently for both IDeg and IGlar across trials, thus eliminating any potential bias because of the differences in algorithms. Further, both the rate and incidence of hypoglycaemia with IGlar in this meta-analysis were consistent with that reported in other trials 26–28, including the Treat-to-Target trial, in which overall confirmed and nocturnal confirmed hypoglycaemic episodes were reported at the respective rates of 3.0 and 1.3 episodes per PYE in insulin-naïve subjects treated with IGlar, respectively 7. The rates reported with IDeg-treated insulin-naïve subjects in this meta-analysis were similar to or lower than the rates reported with insulin detemir (IDet) (3.67 minor and 0.70 nocturnal minor episodes per PYE, respectively) added on to oral antidiabetic drugs in insulin-naïve patients in a treat-to-target trial 6 (the plasma glucose cut-off for minor episodes was 3.1 mmol/l). The same trial also evaluated hypoglycaemia with neutral protamine Hagedorn (NPH) insulin (7.14 and 1.77 episodes per PYE, respectively) 6; these rates were higher than that with IDet, IGlar or IDeg. A systematic Cochrane review of randomized clinical trials in T2DM patients found that, compared with NPH insulin, the risk of experiencing a severe hypoglycaemic episode was 30% lower with IGlar and 50% lower with IDet, the risk of experiencing of a nocturnal hypoglycaemic episode was 34% lower with IGlar and 37% lower with IDet, and the risk of experiencing any hypoglycaemic episode was 18% lower with IDet 29. The Cochrane study concluded that the rates of symptomatic, overall and nocturnal hypoglycaemia were statistically significantly lower in patients treated with IDet or IGlar than those treated with NPH insulin 29, showing the progressive reduction of the risk of hypoglycaemia from NPH insulin to IGlar and IDet—a progression that will be continued with IDeg. (It should be noted that the reporting of hypoglycaemia in randomized controlled trials in the literature has not followed a consistent approach, and the definition of hypoglycaemia varies across trials 29,30).

Although the exclusion of subjects with a history of recurrent severe hypoglycaemia from these trials may be another limitation, this criterion was enforced because the patients with hypoglycaemic unawareness (indicated by recurrent severe hypoglycaemia) may be unsuitable candidates for treatment to the glycaemic levels recommended for the general diabetes population. Further, inclusion of these patients could have introduced heterogeneity to this meta-analysis.

In conclusion, hypoglycaemia is widely acknowledged as the main limiting factor to achieving tight glycaemic control. This pre-planned meta-analysis shows that similar improvements in HbA1c can be achieved with fewer hypoglycaemic episodes, particularly nocturnal episodes, with IDeg than with IGlar across a broad spectrum of patients with diabetes (particularly T2DM patients), and insulin regimens. The benefits of lower rates of nocturnal hypoglycaemia with IDeg over that of IGlar are most clearly demonstrated for both T1DM and T2DM patients during the maintenance phase of their treatment once the optimal dosage has been determined. The lower rates of hypoglycaemia observed with IDeg than with IGlar across individual trials, and the support of these findings in this meta-analysis, provide strong evidence of the benefits of IDeg in reducing the risk of hypoglycaemia in patients with diabetes, thus allowing safer and more intensive insulin titration to minimize diabetes complications.

## Appendix

**Table A1:** Summary of trials included in the meta-analysis

Trial	Trial description and treatment	Population	Antidiabetic therapy at screening	Duration (weeks)	Rand ratio	Number of subjects
3579	IDeg OD vs. IGlar OD (+ met ± DPP-4I)	T2DM insulin-naïve	Met monotherapy or Met + (SU ± α-GI ± DPP-4I in any combination)	52	3:1	IDeg: 773 IGlar: 257
3672	IDeg 200 U/mL OD vs. IGlar OD (+ met ± DPP-4I)	T2DM insulin-naïve	Met monotherapy or Met + (SU/Glin ± α-GI ± DPP-4I in any combination)	26	1:1	IDeg: 228 IGlar: 229
3586	IDeg OD vs. IGlar OD (+ OAD except DPP-4I)	T2DM insulin-naïve	Monotherapy (met or SU) or met + (SU ± α-GI ± DPP-4I) or SU + (α-GI ± DPP-4I) or met + SU + (α-GI or DPP-4I)	26	2:1	IDeg: 289 IGlar: 146
3668	IDeg Flex vs. IGlar OD and IDeg Flex vs. IDeg OD (all arms ± OADs according to label)	T2DM insulin-naïve or basal-insulin-treated	OAD(s) only (any combination of met ± SU/glin ± PIO) or basal insulin only or basal insulin + OADs	26	1:1:1	IDeg FF: 229* IDeg: 228 IGlar: 230
3582	IDeg OD vs. IGlar OD (+ IAsp TID ± met ± PIO)	T2DM insulin-treated	Any insulin regimen (with or without OADs)	52	3:1	IDeg 744 IGlar: 248
3583	IDeg OD vs. IGlar OD (+ IAsp TID)	T1DM insulin-treated	Any basal-bolus regimen	52	3:1	IDeg: 472 IGlar: 157
3770	IDeg Flex vs. IGlar OD and IDeg Flex vs. IDeg OD (all arms + IAsp TID)	T1DM insulin-treated	Any basal insulin (OD or BID) + any bolus injection (≥3 daily injections)	26	1:1:1	IDeg FF: 164* IDeg: 165 IGlar: 164

α-GI, alpha glucosidase inhibitor; BID, twice daily; DPP-4I, dipeptidyl peptidase 4 inhibitor; FF, fixed flexible (flexible dosing arm); Flex, flexible; Glin, glinides; IAsp, insulin aspart; IDeg, insulin degludec; IGlar, insulin glargline; OAD, oral antidiabetic drug; OD, once daily; met, metformin; PIO, pioglitazone; Rand ratio, randomization ratio; SU, sulfonylureas; TID, thrice daily; T2DM, type 2 diabetes mellitus; T1DM, type 1 diabetes mellitus.

* The flexible dosing arms of Trials 3668 and 3770 were excluded from this meta-analysis as this regimen does not reflect the intended use of IDeg in clinical practice.

**Table A2:** Demographics and baseline characteristics of subjects

Trial	3579		3672		3586		3668		3582		3583		3770	
Population	T2DM		T2DM		T2DM		T2DM		T2DM		T1DM		T1DM	
Treatment	IDeg*	IGlar*	IDeg*	IGlar*	IDeg*	IGlar*	IDeg*	IGlar*	IDeg*	IGlar*	IDeg*	IGlar*	IDeg*	IGlar*
No. of subjects	773	257	228	229	289	146	228	230	744	248	472	157	165	164
Sex, N (%), male	471	167	119	124	158	75	124	111	405	133	278	90	94	88
	(60.9)	(65.0)	(52.2)	(54.1)	(54.7)	(51.4)	(54.4)	(48.3)	(54.4)	(53.6)	(58.9)	(57.3)	(57.0)	(53.7)
Age, years, mean (SD)	59.3	58.7	57.8	57.3	58.8	58.1	56.5	56.7	59.2	58.1	42.8	43.7	44·.5	44.1
	(9.7)	(9.9)	(9.0)	(9.4)	(9.8)	(10.1)	(9.6)	(8.8)	(9.1)	(10.0)	(13.7)	(13.3)	(13.1)	(12.6)
Region, N (%)														
Asia	0	0	0	0	200	102	60	64	18	5	0	0	0	0
					(69.2)	(69.9)	(26.3)	(27.8)	(2.4)	(2.0)				
Europe	394	137	85	84	0	0	121	130	317	107	119	39	77	80
	(51.0)	(53.3)	(37.3)	(36.7)			(53.1)	(56.5)	(42.6)	(43.2)	(25.2)	(24.8)	(46.7)	(48.8)
Japan	0	0	0	0	89	44	0	0	0	0	0	0	0	0
					(30.8)	(30.1)								
North America	379	120	136	134	0	0	0	0	377	123	328	111	88	84
	(49.0)	(46.7)	(59.6)	(58.5)					(50.7)	(49.6)	(69.5)	(70.7)	(53.3)	(51.2)
South Africa	0	0	7	11	0	0	17	12	32	13	25	7	0	0
			(3.1)	(4.8)			(7.5)	(5.2)	(4.3)	(5.2)	(5.3)	(4.5)		
South America	0	0	0	0	0	0	30	24	0	0	0	0	0	0
							(13.2)	(10.4)						
Antidiabetic therapy, N (%)														
Insulin ± OAD	0	0	0	0	0	0	97	96	744	248	472	157	16	164
							(42.5)	(41.7)	(100.0)	(100.0)	(100.0)	(100.0)	(100.0)	(100.0)
Insulin glargine							41	30	322	105	336	108	107	100
							(42.3)†	(31.3)†	(43.3)	(42.3)	(71.2)	(68.8)	(64.8)	(61.0)
OAD only	773	257	228	229	289	146	131	134	0	0	0	0	0	0
	(100.0)	(100.0)	(100.0)	(100.0)	(100.0)	(100.0)	(57.5)	(58.3)						
Diabetes duration, years, mean (SD)	9.4	8.6	8.4	8.0	11.8	11.1	10.3	10.8	13.6	13.4	19.1	18.2	20.0	18.2
	(6.3)	(5.7)	(6.7)	(5.6)	(6.5)	(6.5)	(6.7)	(6.4)	(7.4)	(6.9)	(12.2)	(11.4)	(12.5)	(11.9)
BMI, kg/m^2^, mean (SD)	30.9	31.6	32.2	32.7	24.6	25.8	29.4	30.0	32.3	31.9	26.3	26.4	26.4	26.8
	(4.8)	(4.4)	(5.4)	(5.3)	(3.4)	(3.7)	(4.9)	(4.7)	(4.7)	(4.5)	(3.7)	(4.2)	(4.0)	(4.0)

BMI, body mass index; OAD, oral antidiabetic drug; OD, once daily; N, number of subjects; SD, standard deviation; T2DM, type 2 diabetes mellitus; T1DM, type 1 diabetes mellitus.

* IDeg and IGlar were both injected once-daily.

† This is the proportion of previous insulin users taking IGlar.

**Table A3:** Hypoglycaemia risk in the pooled type 2 and type 1 diabetes mellitus population

	Estimated Rate Ratio* (95% Confidence Interval)
Population	Pooled T2DM and T1DM
Number of subjects	
IDeg†	2899
I Glar†	1431
Overall confirmed hypoglycaemic episodes	
Entire treatment period	0.91 [0.83;0.99]‡
Titration period	1.00 [0.90;1.10]
Maintenance period	0.84 [0.75;0.93]‡
Nocturnal confirmed hypoglycaemic episodes	
Entire treatment period	0.74 [0.65;0.85]‡
Titration period	0.86 [0.74;1.00]‡
Maintenance period	0.68 [0.58;0.80]‡
Severe hypoglycaemic episodes	
Entire treatment period	0.98 [0.66;1.45]

IDeg, insulin degludec; IGlar, insulin glargine; T2DM, type 2 diabetes mellitus; T1DM, type 1 diabetes mellitus.

* Estimated rate ratio: IDeg/IGlar.

† IDeg and IGlar were both injected once daily.

‡ Significantly lower risk with insulin degludec based on 95% confidence interval.

**Table A4:** Clinical implications

The results of the individual trials show that patients who initiate with IGlar as basal insulin therapy can, after initial titration, expect around 1.8 overall confirmed hypoglycaemic episodes per year, of which 0.4 episodes per year occur during the night (Table 1, Trial 3579; entire treatment period); hence, for every three subjects initiated and treated with IDeg instead of IGlar for 1 year, one overall confirmed episode will be avoided (calculated using the estimated 17% reduction in the pooled T2DM population across the entire treatment period). Likewise, for every eight subjects treated with IDeg instead of IGlar for 1 year, one nocturnal confirmed episode will be avoided (calculated using the estimated 32% reduction in the pooled T2DM population for the entire treatment period). In other words, for every 100 subjects treated with IDeg instead of IGlar for 1 year, a total of 33 overall confirmed episodes, of which 13 are nocturnal confirmed episodes, are avoided.
The corresponding numbers for T2DM patients on a basal–bolus regimen are not surprisingly much higher: for every 100 subjects treated with IDeg instead of IGlar for 1 year, a total of 232 overall confirmed episodes, of which 59 are nocturnal episodes, are avoided (calculated using the observed rates following treatment with IGlar for the entire treatment period [Table 1; Trial 3582] and the estimated 17% reduction in overall confirmed episodes and 32% reduction in nocturnal confirmed episodes in the pooled T2DM population across the entire treatment period). Hence the clinical relevance of the reduction in hypoglycaemic rates should be interpreted with the progressive nature of T2DM in mind.

## References

[1] Nathan DM, Cleary PA, Backlund JY (2005). Intensive diabetes treatment and cardiovascular disease in patients with type 1 diabetes. N Engl J Med.

[2] UK Prospective Diabetes Study (UKPDS) Group (1998). Intensive blood-glucose control with sulphonylureas or insulin compared with conventional treatment and risk of complications in patients with type 2 diabetes (UKPDS 33). Lancet.

[3] Brod M, Christensen T, Thomsen TL, Bushnell DM (2011). The impact of non-severe hypoglycemic events on work productivity and diabetes management. Value Health.

[4] Bron M, Marynchenko M, Yang H, Yu AP, Wu EQ (2012). Hypoglycemia, treatment discontinuation, and costs in patients with type 2 diabetes mellitus on oral antidiabetic drugs. Postgrad Med.

[5] Cryer PE (2002). Hypoglycaemia: the limiting factor in the glycaemic management of Type I and Type II diabetes. Diabetologia.

[6] Hermansen K, Davies M, Derezinski T, Martinez RG, Clauson P, Home P (2006). A 26-week, randomized, parallel, treat-to-target trial comparing insulin detemir with NPH insulin as add-on therapy to oral glucose-lowering drugs in insulin-naive people with type 2 diabetes. Diabetes Care.

[7] Riddle MC, Rosenstock J, Gerich J (2003). The treat-to-target trial: randomized addition of glargine or human NPH insulin to oral therapy of type 2 diabetic patients. Diabetes Care.

[8] Cryer PE (2008). The barrier of hypoglycemia in diabetes. Diabetes.

[9] Jonassen I, Havelund S, Hoeg-Jensen T, Steensgaard DB, Wahlund PO, Ribel U (2012). Design of the novel protraction mechanism of insulin degludec, an ultra-long-acting basal insulin. Pharm Res.

[10] Heise T, Nosek L, Bottcher SG, Hastrup H, Haahr H (2012). Ultra-long-acting insulin degludec has a flat and stable glucose-lowering effect in type 2 diabetes. Diabetes Obes Metab.

[11] Heise T, Hermanski L, Nosek L, Feldman A, Rasmussen S, Haahr H (2012). Insulin degludec: four times lower pharmacodynamic variability than insulin glargine under steady-state conditions in type 1 diabetes. Diabetes Obes Metab.

[12] Birkeland KI, Home PD, Wendisch U (2011). Insulin degludec in type 1 diabetes: a randomized controlled trial of a new-generation ultra-long-acting insulin compared with insulin glargine. Diabetes Care.

[13] Zinman B, Fulcher G, Rao PV (2011). Insulin degludec, an ultra-long-acting basal insulin, once a day or three times a week vs. insulin glargine once a day in patients with type 2 diabetes: a 16-week, randomised, open-label, phase 2 trial. Lancet.

[14] Bergenstal R, Bhargava A, Jain RK, Unger J, Rasmussen S, Mersebach H, Gough S

[15] Garber AJ, King AB, Del Prato S (2012). Insulin degludec, an ultra-longacting basal insulin, versus insulin glargine in basal–bolus treatment with mealtime insulin aspart in type 2 duabetes (BEGIN Basal-Bolus Type 2): a phase 3, randomised, open-label, treat-to-target non-inferiority trial. Lancet.

[16] Heller S, Buse J, Fisher M (2012). Insulin degludec, an ultra-longacting basal insulin, versus insulin glargine in basal-bolus treatment with mealtime insulin aspart in type 1 duabetes (BEGIN basal-bolus type 1): a phase 3, randomised, open-label, treat-to-target non-inferiority trial. Lancet.

[17] Mathieu C, Hollander P, Lane W (2012). Insulin degludec allows for flexible daily dosing in type 1 diabetes, providing equal glycemic control with less nocturnal hypoglycemia than insulin glargine over 52 weeks. Diabetes.

[18] Onishi Y, Park S, Yoo S, Clauson P, Tamer S, Iwamoto Y (2012). Insulin degludec improves glycemic control in insulin-naïve patients with type 2 diabetes: results of a randomized pan-Asian trial. Diabetes.

[19] Russell-Jones D, Hollander P, Miranda-Palma B (2012). Altering the time of day of once-daily dosing of insulin degludec achieves similar glycemic control and safety compared to dosing the same time of day in people with type 1 diabetes. Diabetes.

[20] Zinman B, Philis-Tsimikas A, Cariou B (2012). Insulin degludec versus insulin glargine in insulin-naive patients with type 2 diabetes: a 1-year, randomized, treat-to-target trial (BEGIN Once Long). Diabetes Care.

[21] Meneghini L, Atkin SL, Bain S (2011). Flexible once-daily dosing of insulin degludec does not compromise glycemic control or safety compared to insulin glargine given once daily at the same time each day in people with type 2 diabetes. Diabetes.

[22] Food and Drug Administration (FDA) http://www.fda.gov/Drugs/GuidanceComplianceRegulatoryInformation/Guidances/ucm064981.html.

[23] Heise T, Hermanski L, Nosek L (2010). Insulin degludec: less pharmacodynamic variability than insulin glargine under steady state conditions. Diabetologia.

[24] Jonassen I, Havelund S, Ribel U (2010). Insulin degludec is a new generation ultra-long acting basal insulin with a unique mechanism of protraction based on multi-hexamer formation. Diabetes.

[25] Turner RC, Cull CA, Frighi V, Holman RR (1999). Glycemic control with diet, sulfonylurea, metformin, or insulin in patients with type 2 diabetes mellitus: progressive requirement for multiple therapies (UKPDS 49). UK Prospective Diabetes Study (UKPDS) Group. JAMA.

[26] Heller S, Koenen C, Bode B (2009). Comparison of insulin detemir and insulin glargine in a basal-bolus regimen, with insulin aspart as the mealtime insulin, in patients with type 1 diabetes: a 52-week, multinational, randomized, open-label, parallel-group, treat-to-target noninferiority trial. Clin Ther.

[27] Hollander P, Cooper J, Bregnhoj J, Pedersen CB (2008). A 52-week, multinational, open-label, parallel-group, noninferiority, treat-to-target trial comparing insulin detemir with insulin glargine in a basal-bolus regimen with mealtime insulin aspart in patients with type 2 diabetes. Clin Ther.

[28] Swinnen SG, Dain MP AR (2010). A 24-week, randomized, treat-to-target trial comparing initiation of insulin glargine once-daily with insulin detemir twice-daily in patients with type 2 diabetes inadequately controlled on oral glucose-lowering drugs. Diabetes Care.

[29] Horvath K, Jeitler K, Berghold A (2007). Long-acting insulin analogues versus NPH insulin (human isophane insulin) for type 2 diabetes mellitus. Cochrane Database Syst Rev.

[30] Little S, Shaw J, Home P (2011). Hypoglycemia rates with basal insulin analogs. Diabetes Technol Ther.

[31] EMEA-Committee for Proprietary Medicinal Products

